# Organ-Based Accumulation, Translocation, and Associated Health Risk of Al, Ni, and Zn in Tomatoes, Peppers, Eggplants, Cucumbers, and Corn from an Industrial Zone in Düzce, Türkiye

**DOI:** 10.3390/foods15020196

**Published:** 2026-01-06

**Authors:** Harun Demirci, Hakan Sevik, Ismail Koc, Handan Ucun Ozel, Ramazan Erdem, Fatih Adiguzel, Erol Imren, Halil Baris Ozel

**Affiliations:** 1 Department of Sustainable Agriculture and Natural Plant Resources, Graduate School, Kastamonu University, 37150 Kastamonu, Türkiye; demirciharun37@hotmail.com; 2 Department of Environmental Engineering, Kastamonu University, 37150 Kastamonu, Türkiye; 3 Department of Forest Engineering, Düzce University, 81620 Düzce, Türkiye; ismailkoc@duzce.edu.tr; 4 Department of Environmental Engineering, Bartin University, 74100 Bartın, Türkiye; handanucun@bartin.edu.tr; 5 Arac Rafet Vergili Vocational School, Kastamonu University, 37800 Kastamonu, Türkiye; rerdem@kastamonu.edu.tr; 6 Department of Transportation Services, Vocational School of Technical Sciences, Bitlis Eren University, 13100 Bitlis, Türkiye; fadiguzel@beu.edu.tr; 7 Department of Forest Engineering, Bartın University, 74100 Bartın, Türkiye; eimren@bartin.edu.tr (E.I.); halilbarisozel@gmail.com (H.B.O.)

**Keywords:** heavy metals, bioconcentration factor, hazard index, target hazard quotient translocation factor

## Abstract

Heavy metals are among the most hazardous pollutants to human health and can be particularly harmful when inhaled or ingested. Therefore, the concentrations of heavy metals in fruits and vegetables grown in regions with high levels of heavy metal pollution should be carefully examined. This study investigated the variation in aluminum (Al), nickel (Ni), and zinc (Zn) concentrations by species and organ in tomatoes, peppers, eggplants, cucumbers, and corn grown near the industrial zone in Düzce, a heavily polluted city in Europe. We determined bioconcentration factors (BCFs) and translocation factors (TFs) in plant organs and assessed the health risk through the Target Hazard Quotient (THQ) and Hazard Index (HI). The results show that Al pollution in the region significantly exceeded the World Health Organization (WHO) and European Union (EU) limit values, and accumulated in all plant organs, including fruits. Furthermore, high levels of metals were translocated from the soil into the organs of peppers and tomatoes. The HI indicated a potential non-carcinogenic health risk (HI > 1) from the consumption of tomatoes, cucumbers, and peppers, primarily driven by Ni. Based on these results, it is recommended that local authorities address Al pollution in the region, avoiding the cultivation of tomatoes and peppers and instead cultivating corn and eggplant. We also observed that Zn levels were very high in the aerial parts of the plants, reaching up to 90% compared to Ni and Al. This study underscores the need to reduce Zn absorption rates, as dietary intake can pose a significant threat to human health.

## 1. Introduction

In the ecosystem, plants perform various ecological, economic, and social roles, most of which are studied for human nutrition. From many points of view, plants sustain life on Earth, but the most discussed is their substantial contribution of nutrients to humans and animals [1,2,3]. Plant development is shaped by the interaction of genetic structure and environmental factors [4,5]. Among the environmental factors, the most important are climatic [6,7] and edaphic [8]. These factors not only determine plant development but also its nutritional content. Various environmental elements can accumulate in plant organs in proportion to their concentrations in the environment. However, this accumulation occurs at different levels in each plant and its organs [9,10,11]. Even so, the nutrient cycle is strongly influenced by toxic metals, particularly in heavily industrialized areas, thereby affecting plant growth and resilience under current climate-heating conditions [12].

Toxic metals, along with essential elements, accumulate in plant organs through soil and water, threatening nutritional beneficence, dietary intake, and human health [13,14,15]. Heavy metals not only influence the health and development of plants but also can have a significant impact on the health of living beings, particularly human consumers. Some heavy metals pose hazards and are carcinogenic to humans, even at low levels. Conversely, essential nutrients can become harmful if present in high concentrations [9,16,17]. Certain elements, in particular, necessitate prioritized evaluation owing to their potential hazards. Among all, Al, Ni, and Zn are listed as priority pollutants by international entities such as the Environmental Protection Agency (EPA) and the Agency for Toxic Substances and Disease Registry (ATSDR). While the EPA has included Ni and Zn, the ATSDR has also included Al [18,19,20].

Aluminum (Al) is prevalent in the environment and constitutes the second most abundant element within the Earth’s crust, accounting for approximately 8% [21]. Aluminum concentrations in the environment are persistently increasing due to anthropogenic activities, including coal combustion, mining operations, waste incineration, and motor vehicle emissions [22]. Nickel (Ni) is found in rocks both as a free metal and in complexes with other metals such as iron. Ni is released into the environment primarily through human activities, including the burning of fossil fuels, vehicle emissions, mining, smelting, and electroplating [23]. Zinc (Zn) is an essential micronutrient that plays a role in various physiological functions of plants. It plays structural and/or catalytic roles in various processes, including cell division, cell expansion, and protein synthesis [24,25]. However, at elevated concentrations, it poses a risk to both plant life and human health. Zn is a metal widely used in the galvanization of steel products, the manufacture of brass items, and the production of casting molds. Activities such as mining and zinc production, waste incineration, fossil fuel combustion, and the use of brake pads, tire components, and agricultural fertilizers contribute to the release of zinc into the environment. The substantial increase in zinc usage and its subsequent environmental release have risen markedly in recent years. In fact, it is reported that Zn production has increased approximately 60-fold from the late 19th century to the present [26].

Due to their potential hazards to human health, determining the concentrations of these elements, especially in air and food, is of great importance. These elements can be very hazardous to health when inhaled or absorbed directly into the human body through food [9,27,28]. Therefore, it is very important to determine the concentrations of these elements in vegetables grown and consumed as food in areas with high levels. Thus, the main objective of the present study was to evaluate the levels of the highly hazardous metals Al, Ni, and Zn in the organs of five human-consumed vegetables (corn, cucumber, eggplant, pepper, and tomato) grown in polluted areas. The origin of the metallic content is known to have resulted from industrialization and urbanization, previously determined at high levels in forest tree species. Thus, in the present human dietary plants, we evaluated accumulation and translocation factors from soil to plant organs to assess whether consumed organs are hyperaccumulators of toxic metals. The final purpose of the study was to determine whether organs of different plants exhibit similar abilities to assimilate toxic Al, Ni, and Zn. The present study will inform future plant management cultivation strategies in the region, as significant land is used around the city for this purpose.

## 2. Materials and Methods

The study was conducted on vegetables grown in areas near an industrial zone in Düzce province. Düzce is one of the most polluted cities in Türkiye, and according to the 2021 World Air Pollution Report, it is among the top five most polluted cities in Europe [28,29]. Previous studies in the region have identified very high concentrations of Al [30], Ni [31], and Zn [32]. In 2023, Düzce exceeded the WHO PM2.5 guideline value of 15 µg/m^3^ over 24 h on 305 days. Düzce is the second-highest elevation point in Türkiye [33]. Studies reveal that the high levels of these heavy metals are related to both the region’s traffic load and industrial activities, as well as its topography. Düzce, where the study was conducted, is at a crossroads in the transportation network connecting many cities to Istanbul, and the city center’s population has increased 3.25-fold in the last 20 years [34]. The increase in population has led to the intertwining of residential, industrial, and agricultural areas.

Five vegetables (tomatoes, peppers, eggplant, corn, and cucumber) were grown for the study. The vegetables, grown from seed in a greenhouse, were planted in the Düzce industrial zone in mid-May. Only irrigation and maintenance (weed control by hand) were performed throughout the growing season. No fertilization, hormone, or pesticide applications were applied during the experiment period. At the end of August, the plants were uprooted and brought to the laboratory. Here, the roots were first cleaned from the soil, the soil was sieved to remove debris, and the roots were placed in glass Petri dishes. The plants were then separated into their individual organs, including roots, stems, leaves, and fruits. The organs were thoroughly washed, rinsed in distilled water, and dissected with steel blades before being transferred to glass Petri dishes. The collected soil samples underwent the same drying and digestion procedures as the plant organs described below.

The samples were stored in the laboratory for 15 days, with the Petri dishes open, in a well-ventilated environment away from direct sunlight. They were then dried in an oven at 45 ± 2 °C for two weeks and ground to a powder using a steel blender. 0.5 g of the samples was weighed and placed in specially designed microwave digestion tubes, and 10 mL of 65% HNO_3_ was added. The samples were then digested in the microwave at 280 PSI and 180 °C for 20 min. Deionized water was added to the sample volume to 50 mL, and it was then filtered through filter paper. Al, Ni, and Zn concentrations were determined using a GBC Integra XL-SDS-270 ICP-OES (Inductively coupled plasma-optical emission spectroscopy) instrument (GBC Scientific Equipment Pty Ltd., Melbourne, Australia). The analysis results that did not fit into the calibration chart were used to create different calibration charts at the ppm or ppb level, and the readings were repeated. During the process, a blank sample was used for the Al, Ni, and Zn elements for quality assurance/quality control (QA/QC). Five standards for the accuracy of each element assessment process were used to plot the calibration curve simultaneously. The detection limits for Al, Ni, and Zn were measured at 0.17 ppm, 280 ppb, and 150 ppb, respectively. After determining the element concentrations, the values were multiplied by the dilution factor to convert to ppb or ppm on a dry weight (dw) basis. This pretreatment and heavy metal analysis have been frequently used in the study of plant samples in recent years [35,36,37]. A total of 300 samples (5 vegetable species × 5 seedlings × 4 organs × 3 replications) were taken for element analysis. The obtained data were analyzed using SPSS 21.1 software. Analysis of variance (ANOVA) was applied to the data to determine the F value and error rate. The Duncan test was performed for factors found to have statistically significant differences at a confidence interval of at least 95%. Principal component analysis (PCA) was performed to identify similarities in the assimilation of toxic metal content across plants and organs.

The bioconcentration factor (BCF) and translocation factor (TF) were also calculated in the study. These factors aimed to determine which species and organs accumulated more of Al, Ni, and Zn. The following formulas were used in the calculations [38].

BCF = Organ concentration/Soil concentration(1)

TF = Organ BCF/Root soil BCF(2)

The Estimated Weekly Intake (EDI) was calculated for Al, Ni, and Zn according to the following equation:

(3) $$EDI=\frac{C\times IR}{BW}$$

C: Metal concentration (mg kg^−1^ dw); IR: Daily ingestion rate [Vegetable: 0.345 kg day^−1^ (adult vegetable intake)]; BW: Body weight (70 kg for adult).

The non-carcinogenic risk was estimated by calculating the target hazard quotient (THQ) of pollutants. THQ is the ratio of the determined dose of pollutant and referent dose level (RfD, mg kg^−1^ bw day^−1^). If THQ is less than 1, the pollutant is unlikely to cause an adverse effect.

(4) $$THQ=\frac{E_{F}\times E_{D}\times IR\times C}{RfD\times BW\times AT}$$

EF; Frequency of exposure (365 days year^−1^), ED; Duration of exposure (70 years, adult), IR; Daily intake (kg day^−1^), C; Metal concentration (mg kg^−1^), RfD; Reference dose (mg kg^−1^ day^−1^), BW; Body weight (70 kg), AT; Mean time (EF × ED) were used. HI was calculated as the sum of THQ. In this study, RfD values were taken as follows: Ni = 0.02 mg kg^−1^ day^−1^; Zn = 0.3 mg kg^−1^ day^−1^; Al = 1 mg kg^−1^ day^−1^ [39].

## 3. Results

### 3.1. Change in Al (ppm) Concentration

The results of the ANOVA and Duncan test for changes in Al concentration in vegetables by species and organ are presented in Table 1. Al concentrations were found to vary statistically significantly across all species and organs (*p* < 0.001) (Table 1). The lowest values were in fruits, and the highest values were in roots across all species. Based on organs, the lowest values in organs other than roots are obtained in corn, and the highest values are obtained in tomatoes. Another notable point was that Al concentrations in roots were much higher than in other organs. The lowest Al values in organs, based on average values, were in peppers, and the highest were in corn. Similarly, in soils, the lowest Al values were observed in peppers, while the highest were observed in corn.

The changes in BCF values for Al by species and organs are shown in Table 2. BCF values for Al ranged from 0.002 (corn fruit) to 1.011 (corn root). BCF values were relatively low, except in peppers and corn roots. According to the average values, the lowest BCF values were calculated for fruit as an organ and for cucumber as a species. In contrast, the highest values were calculated in the root as an organ and corn as a species.

The calculated TF values for Al are shown in Table 3. An examination of the TF values for Al reveals that the lowest values were obtained in fruit across all species. The TF values for fruit were calculated as 0.002 in corn. The TF values calculated for other fruit species ranged from 0.010 (eggplant) to 0.023 (cucumber). The highest TF values for all species were observed in leaves, with the highest in tomato (0.704) and cucumber (0.439).

### 3.2. Changes in Ni (ppb) Concentration

The results of the analysis of variance and Duncan’s test for changes in Ni concentration are presented in Table 4. Upon examining the table values, it is evident that Ni concentrations changed significantly in all organs and species (*p* < 0.001). The lowest values were found in the leaves of corn and the fruits of eggplant, whereas the highest values were found in the stems of other species. The highest values were also found in the roots of all species. Species-wise, the lowest values were found in the stems and roots of cucumber, the fruits of eggplant, and the leaves of corn. The highest values were found in the stems and roots of corn, and the fruits and leaves of tomato. It is noteworthy that the Ni concentration calculated in corn roots was much higher than in other organs. In soils, the lowest values were observed in peppers and tomatoes, and the highest in eggplant.

The changes in BCF values for Ni by species and organs are presented in Table 5. As shown in Table 5, BCF values for Ni ranged from 0.039 (corn leaves) to 1.111 (corn roots). The lowest values for corn and eggplant were found in the stem, while the lowest values for other vegetables were found in the fruit. The average BCF values varied as follows: cucumber < eggplant < tomato < corn < pepper.

The calculated TF values for Ni are shown in Table 6. The lowest TF value for Ni was found in corn leaves at 0.035, while the highest TF value was found in tomato leaves at 0.643. The lowest TF values were observed in corn, ranging from 0.035 to 0.140. No organ had a TF value exceeding 1, with the highest values being found in tomato leaves (0.643), cucumber leaves (0.516), and pepper leaves (0.488).

### 3.3. Change in Zn (ppb) Concentration

Mean data for changes in Zn concentration, along with the results of analysis of variance and Duncan’s test, are presented in Table 7. As with the other two elements, changes in Zn were statistically significant in all species and organs (*p* < 0.001). The highest values were obtained in eggplant stems, cucumber fruit, pepper leaves, and corn roots. It is noteworthy that the highest values were observed in each organ across different species. Regarding organs, the highest values were obtained in pepper leaves, tomato stems, and roots in other species. Changes in Zn concentration in fruits were tomato < eggplant < pepper < corn < cucumber. Changes in Zn concentration in soils were observed in the following order: pepper < eggplant < cucumber < tomato < corn.

Changes in BCF values for Zn by species and organs are presented in Table 8. BCF values for Zn were determined to range from 0.120 (corn stem) to 1.495 (pepper leaf). According to the calculations, the lowest values were found in the stems of corn and cucumber, as well as in the fruits of other species. The highest values were found in the leaves of pepper, the stems of tomatoes, and the roots of different species.

The calculated TF values for Zn are given in Table 9. An examination of the table values reveals that the TF values for Zn are particularly high in pepper and cucumber. The highest values were found in pepper leaves (3.421), tomato stems (2.233), and pepper stems (1.522). Furthermore, the TF values calculated for pepper fruits (1.217) exceeded 1. The lowest values were found in corn stems (0.141) and corn leaves (0.326).

**Figure 1:**
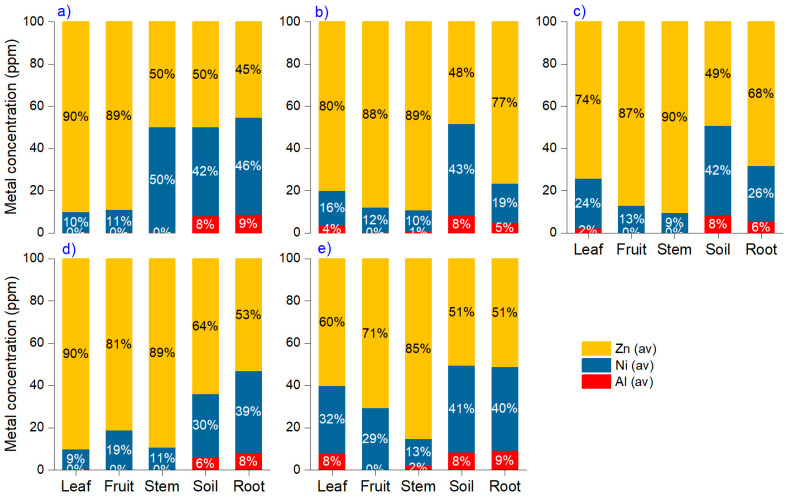
Cumulative percentage of metal content in plant organs: (**a**) corn, (**b**) cucumber, (**c**) eggplant, (**d**) pepper, and (**e**) tomato.

The reduction in metal levels from soil to roots, leaves, stems, and fruit is observed for Al, Ni, and Zn, indicating that plants primarily bioaccumulate these minerals from the soil solution. Leaf and stems are susceptible to atmospheric deposition, but the amounts are much lower than those resulting from soil. According to our findings, Zn is highly available mainly in leaves and fruits, where it can reach 90% compared with Ni and Al (Figure 1). The ratio is similar to that of soil composition, where Zn is around 50%, followed by Ni (~42%) and Al (~8%).

**Figure 2:**
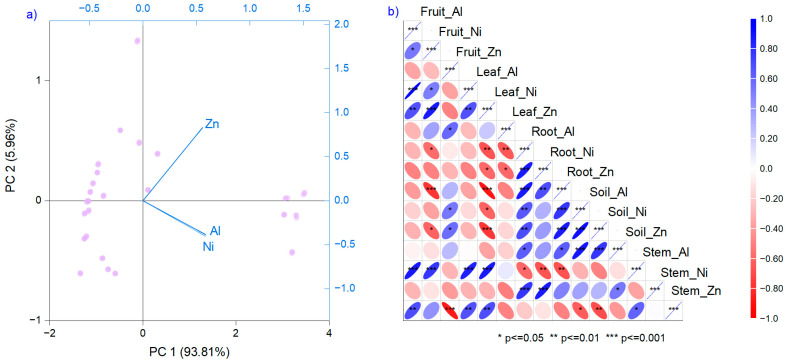
(**a**) Principal component analysis diagram (PCA) and (**b**) correlation analysis between elemental profiles in plant organs.

The relationships between metal content in various matrices, as analyzed by PCA (Figure 2a), and correlation analysis (Figure 2a), indicate strong, significant positive and negative associations, both within plant organs and between metals (Figure 2). Thus, a positive relationship between Al and Ni is observed in fruits and leaves, with values up to *r* = 0.97 (Figure 2b). Ni in leaf is strongly negatively correlated with root (Al, Ni, and Zn) and soil (Al and Ni). Al in roots is positively associated with root and soil metals and negatively associated with stem Al. A different pattern was found for Zn in fruits, which was positively correlated with soil Al and Ni, and leaf Zn was negatively associated with root Al and Ni. These results indicate antagonistic relationships between Zn and Al, and between Zn and Ni, in organs primarily used for human nutrition. Still, the path of metals in fruits is documented to be from the soil. The results are sustained by PCA, which shows a high variance explained through the first principal component (93.81%). In this analysis, Al and Ni are associated with the group with the highest eigenvalues (2.81 and 0.17), followed by Zn (0.007) (Figure 2a).

The calculated THQ values for corn, cucumber, tomato, eggplant, and pepper are given in Table 10. The THQ values revealed that Al and Zn posed negligible non-carcinogenic risk across all vegetable species (THQ < 1). In contrast, Ni showed comparatively higher THQ values, particularly in tomato, cucumber, and pepper fruits. The hazard index (HI) exceeded the safe threshold (HI > 1) for tomato, cucumber, and pepper, suggesting potential non-carcinogenic health risks associated with their consumption.

## 4. Discussion

This study examined the accumulation and translocation of Al, Ni, and Zn in some of the most commonly consumed vegetables. It is known that the elements in question can cause serious health problems when consumed through food. Al can be ingested through the respiratory tract, ingestion, or dermal pathways. Al is a neurotoxic agent and can be absorbed through the gastrointestinal tract and lungs. When ingested, it can disrupt the central nervous system and cause dialysis encephalopathy, osteomalacia, and microcytic anemia [30,40,41].

Additionally, Al accumulation in brain tissue can cause neurological and cognitive disorders [42,43]. It has been determined that the occurrence of Alzheimer’s disease is associated with aluminum, and high levels of aluminum in the body can be transported to the brain through the vascular system, leading to brain inflammation and death [21,30,44]. Aluminum accumulation in the liver has been found to cause cholestasis, shortening the lifespan of blood cells, and is responsible for several hematological changes, including impaired erythropoiesis [42]. Therefore, determining the concentration of Al in plants consumed as food is of great importance.

Al is not an essential element for plants and can cause toxicity when accumulated in high amounts. The WHO permitted concentration of Al is 0.2 mg/L [23]. There is a European Union technical guideline stating a specific release limit of no more than 5 mg of Al per kg of food [45]. The study determined that Al concentrations in fruiting bodies consumed as food ranged from 15.06 ppm (corn) to 44.00 ppm (tomatoes). These values are tens of times higher than the permitted values set by the WHO and the EU.

Studies conducted to date have determined mainly that Al concentrations in cucumbers range from 0.400 to 4.17 mg/kg, with an average of 0.905 mg/kg; in eggplants, from 0.500 to 1.50 mg/kg, with an average of 0.800 mg/kg; in peppers, from 0.500 to 3.30 mg/kg, with an average of 1.26 mg/kg; and in corn, from 0.115 to 0.250 mg/kg, with an average of 0.175 mg/kg [45]. A study conducted in Kastamonu found average Al concentrations of 39,800 μg kg^−1^ dw in eggplant, 796 μg kg^−1^ dw in tomatoes, 4997 μg kg^−1^ dw in cucumbers, and 11,452 μg kg^−1^ dw in peppers [46]. Compared to these values, the Al concentrations obtained in the study are much higher.

Another element evaluated in the study is Ni. Ni is one of the most hazardous elements to human health, and the International Agency for Research on Cancer (IARC) has classified Ni compounds as Group 1 (carcinogenic to humans) and Ni and its alloys as Group 2B (possibly carcinogenic to humans). It has been reported that Ni can cause various medical problems, including asthma, cardiovascular diseases, lung fibrosis, contact dermatitis, skin irritation, lung, stomach, and kidney damage, cardiovascular diseases, lung fibrosis, headaches, and gastrointestinal symptoms [18,19,47].

Ni is essential for plants at low concentrations; however, high concentrations cause harmful effects on plant growth [24,48]. However, the Turkish Food Codex does not set an upper limit for Ni for vegetables. The study determined that nickel concentrations in fruits ranged from 1746 ppb (eggplant) to 3885 ppb (tomato). In a study conducted in Iran, Ni concentration was determined to be around 0.007 mg/kg in tomatoes and 0.037 mg/kg in cucumbers [49]. In a study conducted in Türkiye, the average Ni concentrations were 1832 μg kg^−1^ dw in eggplant, 724 μg kg^−1^ dw in cucumber, and 1609 μg kg^−1^ dw in pepper [46]. In a study conducted in Ankara, Türkiye, it was determined that the Ni concentration in washed samples ranged from 1346.2 to 3680.8 ppb in pepper, 433.3 to 1438.4 ppb in tomato, and 1649.0 to 3068.7 ppb in cucumber, depending on traffic density [50].

The high concentration of Ni accumulation in maize roots is directly related to the plant’s defense strategies and ion transport mechanisms. In the scientific literature, maize is described as an “excluder” plant that restricts the transport of Ni to above-ground organs and traps the metal in the root system. Maize possesses a strong physiological barrier system that prevents Ni from passing to the stem and leaves. Roots rapidly absorb nickel ions from the soil through passive diffusion or low-affinity carriers; however, the loading of these metals into the xylem is limited. Studies have shown that in maize, Ni accumulates at the highest concentration in the root apex and the endodermis layer. The endodermis acts as a “security wall,” preventing nickel from passing into the central cylinder and thus to the stem [51].

The TF values for Ni in maize have generally been found to be well below 1. This situation arises because HMA (Heavy Metal ATPase) type proteins or chelators, which enable the loading of Ni into the xylem, have limited capacity for Ni in maize. As a result, a large portion of the absorbed nickel remains in the root tissues. Some of the Ni binds to negatively charged regions (pectins, etc.) in the cell walls of root cells. This apoplastic attachment causes Ni to be physically fixed in the root structure, preventing it from entering the cell. In the literature, it has been reported that the Ni content in maize roots can sometimes be more concentrated in the cell wall than in the protoplasm [52,53,54].

The final element evaluated in the study was zinc. Zn is a toxic element for humans, and symptoms such as nausea, vomiting, diarrhea, loss of appetite, stomach cramps, headaches, weakened immune systems, and indigestion can be observed in cases of Zn toxicity [19,55,56]. The acceptable upper limits of Zn in vegetables have been reported as 50–150 μg/g [57]. The study determined that zinc concentrations in fruits ranged from 9475 ppb (in tomatoes) to 17,458 ppb (in cucumbers). According to these results, the highest value determined in fruits (approximately 17.5 ppm) is well below the permissible upper limits (at least 50 ppm). In a study on the subject, the average Zn concentrations were determined as 8735 μg kg^−1^ dw in eggplant, 1023 μg kg^−1^ dw in tomato, 6120 μg kg^−1^ dw in cucumber, and 73,002 μg kg^−1^ dw in pepper [46]. In a study conducted in Iran, Zn concentrations were determined to be 38.396 mg/kg in tomatoes and 23.440 mg kg^−1^ in cucumbers [49]. In a study conducted in Saudi Arabia, Zn concentrations were 552.77 ± 48.41 μg/g in the outer tissues of pepper and 686.71 ± 94.09 μg/g in the inner tissues. At the same time, they remained below the detectable limits in tomatoes, eggplant, and cucumber [57]. Another study conducted on vegetables collected from markets in Saudi Arabia determined Zn concentrations to be 0.65–3.46 µg/g dw in cucumbers, 50.48 ± 19.00 µg/g dw in tomatoes, 3.90 ± 0.78 µg/g dw in eggplants, and 6.07 ± 1.89 µg/g dw in peppers [58]. Based on these results, it can be stated that the Zn values obtained in this study are consistent with those reported in similar studies.

The higher Zn translocation capacity in pepper plants compared to other vegetable species is directly related to both the activity of specific carrier proteins and the chelation mechanisms within the plant. This capacity enables the rapid and efficient transport of Zn from the roots to the above-ground organs (stems and leaves) via the xylem. The ZIP (ZRT/IRT-like Proteins) and HMA (Heavy Metal ATPase) gene families, which are responsible for Zn transport in pepper species, have been observed to be highly active. In particular, HMA2 and HMA4 proteins play a critical role in xylem loading of Zn ions. The pepper plant actively pumps Zn ions from its root cells into the xylem, maximizing mass flow towards the leaves [59]. Organic acids such as citrate and malate, which are present in high concentrations in pepper leaves, chelate with Zn, preventing the ion from precipitating in the xylem sap and increasing its mobility. Studies show that pepper plants have a high capacity for synthesizing these organic ligands in their stem and leaf tissues, which accelerates their “source-to-sink” transport [60]. The expression level of Nicotianamine Synthase (NAS) genes in pepper plants directly affects the Zn translocation coefficient. Nicotianamine binds to Zn ions, enabling their movement in both the xylem and phloem. Higher NA production compared to other species allows pepper plants to transport more Zn to metabolically active regions, such as fruits and young leaves [61].

For vegetables, legal limits for the elements Ni and Zn in the study have not been determined in detail. However, compared with the limit values established for other harmful elements, the values obtained in the study appear very high. The WHO and EU set limit values for As and Cd in leafy vegetables at 0.1 mg/kg. The WHO set a limit value for Pb of 0.3 mg/kg, and the EU set a limit value of 0.1 mg/kg [62]. The values obtained in the study are well above these limits.

The uptake of heavy metals by vegetables varies depending on soil characteristics, including water, temperature, texture, pH, organic matter content, and nutrient availability, as well as the specific vegetable species [63,64,65]. Furthermore, the potential for plants to accumulate heavy metals is also linked to their phenology and development [66]. Plant phenology and development are shaped by genetic structure [67,68] and environmental conditions [69,70]. In short, different genetic structures or variable environmental conditions in plants can significantly affect phenology, development, and heavy metal uptake. Additionally, many vegetables have subspecies, forms, or cultivars that are genetically differentiated [71,72]. At the same time, the accumulation of heavy metals in plants is directly related to the concentration of these metals in the environment. For these reasons, comparing heavy metal results in plants grown in different environments can lead to misinterpretations.

The study found that soil heavy metal content is relatively high. Heavy metals can enter plants directly from the air through the stomata on their leaves or stems. However, the most important entry route is through the roots [73,74]. High heavy metal concentrations in soil also lead to high concentrations in roots [38,75]. Therefore, the concentrations of heavy metals in soil should be considered. In Türkiye, the accepted limit values for Ni in soils are 75 mg/kg and for Zn are 300 mg/kg [76]. The results of the study indicate that these limit values were not exceeded.

The study found that TF values were generally higher in leaves than in other organs, with the highest average values across all three elements observed in leaves. While TF values for Al and Ni did not exceed 1 in any organ, TF values for Zn were calculated to be above 1 in pepper leaves (3.421), tomato stems (2.233), pepper stems (1.522), and pepper fruits (1.217). The TF value is a crucial indicator of a species’ potential to accumulate heavy metals. The higher this value, the higher the species’ potential for heavy metal accumulation in that organ [77]. Plants with TF values greater than 1 are considered strong accumulators of the relevant metals [78,79]. These results suggest that peppers and tomatoes, among the plants studied, can accumulate Zn strongly in various organs. The substantial Zn accumulation of pepper fruits, in particular, may pose a risk to human health.

## 5. Conclusions and Recommendations

The study found that Al concentrations in vegetables and fruits of all types were significantly above the WHO and EU limit values. The metal concentration in plant organs arrives from the soil, which was also exceptionally high. These results suggest that Al pollution in the region is high and that the crops grown there are affected, posing serious health risks. The health risk assessment (THQ and HI) indicates a potential non-carcinogenic health risk from the consumption of tomatoes, cucumbers, and peppers, with Hazard Index exceeding the safe threshold (HI > 1). Results indicate that the translocation factor for Zn concentration from soil to plant organs, especially in peppers and tomatoes, is high in the region. The correlation analysis reveals both positive and negative relationships between metallic content in plants’ organs and interactions among metals. Furthermore, we observed that Zn in fruits is exceptionally high in almost all plants, up to 90% reported to Al and Ni. The peppers and tomatoes grown in this region contain high levels of Zn, Al, and Ni, which can affect human metabolism even at low levels. While these elements are reported to be highly harmful to human health, limit values for Ni and Zn in vegetables have not yet been established. Therefore, it cannot be determined whether these elements exceed the limit values in the vegetables studied. However, the high concentrations observed are evident compared to those of other elements. Therefore, the limit values of these elements in fruits and vegetables must be determined urgently for health reasons. The Target Hazard Quotient analysis specifically specified Ni as the primary driver of the cumulative health risk in tomatoes, cucumbers, and peppers. In areas with high Zn pollution, it is recommended to grow corn and eggplant, which have much lower TF values in their fruits, rather than these vegetables. Studies have shown a high correlation between heavy metals. The high concentrations of these elements suggest that heavy metals such as As, Pb, Cd, Cu, Cr, Ba, Sr, Tl, Sn, V, and Se, which pose a threat to human health even at very low concentrations, may also be present in the region at high concentrations. Therefore, comprehensive studies are recommended in the region.

## Figures and Tables

**Table 1:** Changes in Al concentration (ppm) in vegetables based on organs.

Species	Organ						
	Stem	Fruit	Leaf	Root	F Value	Mean	Soil
Pepper	42 cB	25 bA	186 bC	1612 aD	112,394.4 ***	466 a	2294 a
Tomato	652 eB	44 eA	1407 eC	2001 cD	45,010.4 ***	1026 b	7409 b
Corn	23 aA	15 aA	34 aA	7859 eB	314,067.4 ***	1983 c	7776 d
Eggplant	38 bB	26 cB	300 cC	2328 dD	470,023.7 ***	673 ab	7398 b
Cucumber	104 dB	38 dA	745 dC	1705 bD	79,868.2 ***	648 ab	7708 c
F value	354,441.9 ***	2271.1 ***	27,600.6 ***	135,021.6 ***		51.5 ***	13,846.8 ***
Mean	172 A	30 A	534 A	3101 B	90.6 ***		

Note: *** = *p* < 0.001. Uppercase letters compare organs within a row (horizontal) for each species, while lowercase letters compare species inside a column (vertical) for each organ. Values with the same letter do not statistically differ.

**Table 2:** BCF values for Al in vegetables.

Species	Organ				Mean
	Stem	Fruit	Leaf	Root	
Pepper	0.018	0.011	0.081	0.703	0.203
Tomato	0.088	0.006	0.190	0.270	0.139
Corn	0.003	0.002	0.004	1.011	0.255
Eggplant	0.005	0.003	0.041	0.315	0.091
Cucumber	0.013	0.005	0.097	0.221	0.084
Mean	0.025	0.005	0.083	0.504	0.154

**Table 3:** TF values for Al in vegetables.

Species	Organ			Mean
	Stem	Fruit	Leaf	
Pepper	0.026	0.016	0.115	0.052
Tomato	0.326	0.022	0.704	0.351
Corn	0.003	0.002	0.004	0.003
Eggplant	0.016	0.010	0.130	0.052
Cucumber	0.059	0.023	0.439	0.174
Mean	0.086	0.015	0.278	0.126

**Table 4:** Changes in Ni concentration (ppb) in vegetables based on organs.

Species	Organ						
	Stem	Fruit	Leaf	Root	F Value	Mean	Soil
Pepper	1874 bA	2914 dB	3722 dC	7611 bD	19,679.6 ***	4030 a	11,098 a
Tomato	3665 cA	3885 eB	5545 eC	8643 cD	6157.8 ***	5434 a	36,293 a
Corn	5797 dC	1784 bB	1456 aA	41,201 eD	55,4309.9 ***	12,559 b	37,074 c
Eggplant	1922 bB	1746 aA	3179 bC	10,990 dD	50,839.9 ***	4459 a	40,400 e
Cucumber	1550 aA	2391 cB	3341 cC	6458 aD	19,460.6 ***	3435 a	40,071 d
F value	6320.6 ***	9283.4 ***	5420.9 ***	221,644.3 ***		2.549 *	20,190.0 ***
Mean	2962 A	2544 A	3448 A	8425 B	47.3 ***		

Note: * = *p* < 0.05; *** = *p* < 0.001. Uppercase letters compare organs within a row (horizontal) for each species, while lowercase letters compare species inside a column (vertical) for each organ. Values with the same letter do not statistically differ.

**Table 5:** BCF values for the Ni element in vegetables.

Species	Organ				Mean
	Stem	Fruit	Leaf	Root	
Pepper	0.169	0.263	0.335	0.686	0.363
Tomato	0.101	0.107	0.153	0.238	0.150
Corn	0.156	0.048	0.039	1.111	0.339
Eggplant	0.048	0.043	0.079	0.272	0.111
Cucumber	0.039	0.060	0.083	0.161	0.086
Mean	0.103	0.104	0.138	0.494	0.210

**Table 6:** TF values for Ni in vegetables.

Species	Organ			Mean
	Stem	Fruit	Leaf	
Pepper	0.246	0.383	0.488	0.372
Tomato	0.424	0.450	0.643	0.506
Corn	0.140	0.043	0.035	0.073
Eggplant	0.176	0.158	0.290	0.208
Cucumber	0.242	0.373	0.516	0.377
Mean	0.246	0.281	0.394	0.307

**Table 7:** Changes in Zn concentration (ppb) in vegetables based on organs.

Species	Organ						
	Stem	Fruit	Leaf	Root	F Value	Mean	Soil
Pepper	15,826 cA	12,666 cB	35,590 eC	10,409 aA	37,483.7 ***	18,623	23,799 a
Tomato	24,845 bD	9475 aA	10,511 bB	11,143 bC	72,276.5 ***	13,993	44,661 c
Corn	5763 aA	14,600 dC	13,255 cB	40,695 eD	81,545.9 ***	18,578	47,869 d
Eggplant	18,346 dC	11,789 bB	9985 aA	28,530 dD	27,634.1 ***	17,163	42,818 b
Cucumber	13,483 bA	17,458 eC	16,320 dB	26,659 cD	17,732.4 ***	18,480	44,608 c
F value	33,009.0 ***	4813.8 ***	33,620.3 ***	65,622.1 ***		0.553 ns	11,728.2 ***
Mean	15,652	13,197	17,132	19,185	1.581 ns		

Note: *** = *p* < 0.001; ns = not significant (*p* > 0.05). Uppercase letters compare organs within a row (horizontal) for each species, while lowercase letters compare species inside a column (vertical) for each organ. Values with the same letter do not statistically differ.

**Table 8:** BCF values for Zn in vegetables.

Species	Organ				Mean
	Stem	Fruit	Leaf	Root	
Pepper	0.665	0.532	1.495	0.437	0.782
Tomato	0.556	0.212	0.235	0.249	0.313
Corn	0.120	0.305	0.277	0.850	0.388
Eggplant	0.428	0.275	0.233	0.666	0.401
Cucumber	0.302	0.391	0.366	0.598	0.414
Mean	0.414	0.343	0.521	0.560	0.460

**Table 9:** TF values for Zn in vegetables.

Species	Organ			Mean
	Stem	Fruit	Leaf	
Pepper	1.522	1.217	3.421	2.053
Tomato	2.233	0.851	0.944	1.343
Corn	0.141	0.359	0.326	0.275
Eggplant	0.643	0.413	0.350	0.469
Cucumber	0.505	0.654	0.612	0.590
Mean	1.009	0.699	1.131	0.946

**Table 10:** THQ values for Al, Ni, and Zn elements, and the HI index values in vegetables.

Vegetables	Al	Ni	Zn	HI
Corn	0.07	0.44	0.24	**0.75**
Cucumber	0.19	0.59	0.29	**1.07**
Tomato	0.22	0.96	0.16	**1.34**
Eggplant	0.13	0.43	0.20	**0.76**
Pepper	0.12	0.72	0.21	**1.05**

## Data Availability

The original contributions presented in the study are included in the article; further inquiries can be directed to the corresponding author.
